# Deep Learning Approaches for Detection of Breast Adenocarcinoma Causing Carcinogenic Mutations

**DOI:** 10.3390/ijms231911539

**Published:** 2022-09-29

**Authors:** Asghar Ali Shah, Fahad Alturise, Tamim Alkhalifah, Yaser Daanial Khan

**Affiliations:** 1 Department of Computer Science, University of Management and Technology, Lahore 54770, Pakistan; 2 Department of Computer, College of Science and Arts in Ar Rass, Qassim University, Ar Rass 58892, Qassim, Saudi Arabia

**Keywords:** breast adenocarcinoma, long short-term memory (LSTM) network, gated recurrent units (GRU), bi-directional LSTM, mutation detection

## Abstract

Genes are composed of DNA and each gene has a specific sequence. Recombination or replication within the gene base ends in a permanent change in the nucleotide collection in a DNA called mutation and some mutations can lead to cancer. Breast adenocarcinoma starts in secretary cells. Breast adenocarcinoma is the most common of all cancers that occur in women. According to a survey within the United States of America, there are more than 282,000 breast adenocarcinoma patients registered each 12 months, and most of them are women. Recognition of cancer in its early stages saves many lives. A proposed framework is developed for the early detection of breast adenocarcinoma using an ensemble learning technique with multiple deep learning algorithms, specifically: Long Short-Term Memory (LSTM), Gated Recurrent Units (GRU), and Bi-directional LSTM. There are 99 types of driver genes involved in breast adenocarcinoma. This study uses a dataset of 4127 samples including men and women taken from more than 12 cohorts of cancer detection institutes. The dataset encompasses a total of 6170 mutations that occur in 99 genes. On these gene sequences, different algorithms are applied for feature extraction. Three types of testing techniques including independent set testing, self-consistency testing, and a 10-fold cross-validation test is applied to validate and test the learning approaches. Subsequently, multiple deep learning approaches such as LSTM, GRU, and bi-directional LSTM algorithms are applied. Several evaluation metrics are enumerated for the validation of results including accuracy, sensitivity, specificity, Mathew’s correlation coefficient, area under the curve, training loss, precision, recall, F1 score, and Cohen’s kappa while the values obtained are 99.57, 99.50, 99.63, 0.99, 1.0, 0.2027, 99.57, 99.57, 99.57, and 99.14 respectively.

## 1. Introduction

Adenocarcinoma is a cancer that begins in secretory cells. The most common types of adenocarcinomas include prostate, lung, breast, pancreatic, colorectal, and stomach cancer. Among all, breast cancer is the second most severe cancer present in the human body. Breast cancer is the uncontrolled growth and abnormality of cells within the breast gland. Breast cancer occurs mostly in women. An expected 0.3 million women are recognized with breast cancers each year inside the United States of America. In 2021, an estimated 44,130 deaths (43,600 women and 530 men) occurred because of breast cancers in the United States [1,2]. There are numerous reasons for breast cancers in women. Some of them are aging, family breast cancer history, having a child after the age of 35, beginning menopause after the age of 55, high bone density, and so forth [1].

**Figure 1:**
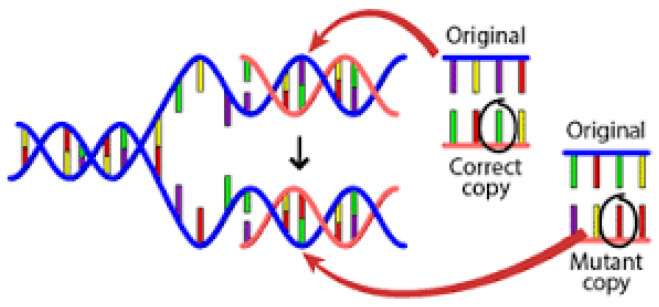
A point mutation in a gene.

A biopsy is the principal technique used to diagnose breast adenocarcinoma. It is a technique in which a small tissue cell is examined with a microscope [3]. The artificial intelligence approach has workable results in the discipline of medical sciences. There are various AI methods used in the medical science area for the detection of several illnesses inside the human body. In this study, the author proposed an ensemble learning strategy that can be employed to become aware of breast cancer at its early stages. There are sequences of DNA in human genes. Any change in the sequence is known as mutation, which in most cases leads to cancer. The procedure of mutation is illustrated in Figure 1 [4].

Genes coordinate with each other by having specific sequences within a cell [5]. Mutation is caused by a change in the base sequence of a DNA. The main cause of this change can also be via insertion, deletion, or replication of gene bases, which causes DNA damage. Different factors influence DNA damage. These factors consist of metabolic influences or environmental factors such as radiation that led to tens of instances of damage per cell, every day [6]. The damage in the DNA molecule alters or eliminates the cell’s capability to transcribe the gene. DNA repair is the procedure in which a cell identifies and corrects the damage that happens in DNA [7]. This technique is constantly energetic as it responds to damage in the DNA structure. When the regular repair process fails, or cellular apoptosis does not occur, then DNA damage may additionally now not be repairable. This irreparable injury leads to malignant tumors, or cancer [8,9].

In this study, deep learning approaches such as LSTM, GRU, and bi-directional LSTM are employed to form a classification mechanism that provides excellent results. The proposed learning approach demonstrated good performance as discussed in the result section.

The second-major cause of death in women is breast adenocarcinoma. Bioinformatics plays a crucial role in the field of medical sciences. Computational technologies, deep learning, and machine learning algorithms make the detection and prevention of diseases much easier than earlier. In this section, some of these techniques that are used for the detection of breast adenocarcinoma are explained.

The most-used machine learning algorithms developed for breast cancer detection are SVM (Support Vector Machine), LR (Logical Regression), RF (Random Forest), MLP (Multilayer Perceptron), and KNN (K-Nearest Neighbor). In [10], breast cancer data are classified using k-Nearest Neighbors, Naïve Bayes, and Support Vector Machine trained and investigated on the WEKA tool. The dataset is taken from the UCI website. For the study, the Radial basic kernel proves the best accuracy of 96.85% for data classification.

Data mining techniques are used to predict and resolve breast cancer survivability [11]. Simple Logistic Regression, Decision Tree, and RBF network are used in this research and the results are validated using a 10-fold cross-validation test. For feature extraction, simple Logistic Regression outperformed all others. The dataset used in this study is taken from a database of the University Medical Centre, Institute of Oncology. Weka is used to train the models. Simple logistics obtained the highest accuracy of 74.47%. In [12] artificial neural networks, decision trees, and logistic regression are used. The accuracy obtained by logistic regression was 89.2%, ANN has an accuracy of 91.2% and the best accuracy was obtained by a decision tree with 93.6%. In [13], the fast correlation-based filter (FCBF) method is used for the prediction and classification of breast cancer. Five machine learning algorithms are applied, including RF, SVM, KNN, Naive Bayes, and MLP. The highest accuracy obtained by SVM is 97.9%. Many other researchers worked on breast cancer identification, as discussed in [14].

## 2. Results

Subsequent paragraphs show the results obtained using different ensemble learning techniques along with various tests.

### 2.1. Self-Consistency Testing

The self-consistency test is the first testing technique used for testing deep learning algorithms for the identification of breast adenocarcinoma. In the self-consistency test complete dataset is used for training and testing purpose. This test ensures that the algorithm will give its best results when it uses all its data for training purposes. The test computes the results of the proposed algorithm without experimentally measuring the stability values [15,16]. The proposed study measured accurate prediction of change in gene sequences in breast adenocarcinoma from the dataset utilizing the protocols based on self-consistency.

Results of proposed ensemble learning model with self-consistency test are discussed in Table 1. The independent set test results are discussed in Table 2 and 10-fold cross validation results are discussed in Table 3.

**Figure 2:**
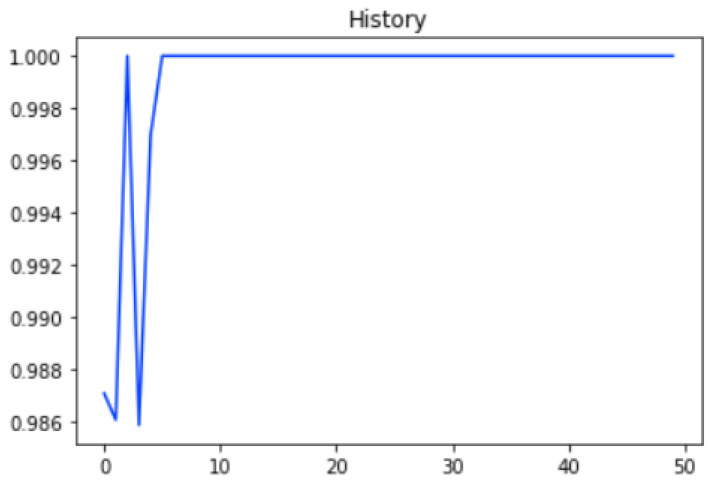
Training history of proposed ensemble model with self-consistency test.

The graph in Figure 2 shows the Training history of the proposed model in self-Consistency test.

**Figure 3:**
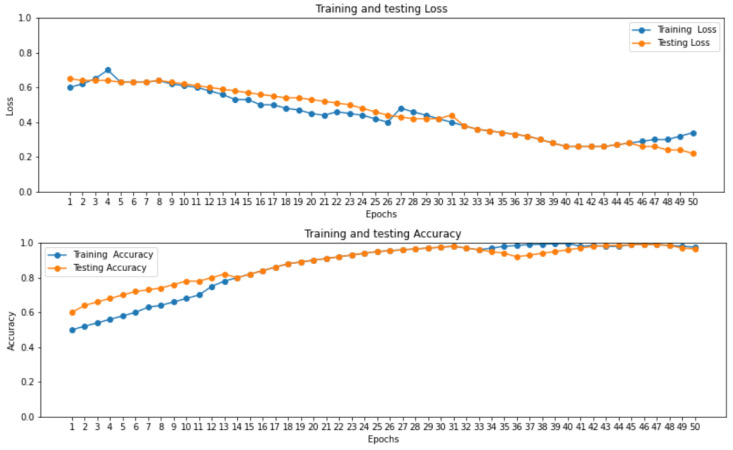
Loss and Accuracy curve of proposed ensemble learning model with Self consistency test.

The accuracy and loss curve of proposed ensemble learning approach for individual deep learning algorithm such as LSTM, GRU, and bi-directional LSTM in self-consistency test is shown in Figure 3.

Figure 3 illustrates that the accuracy of the proposed ensemble learning approach for individual deep learning algorithm such as LSTM, GRU, and bi-directional LSTM is increasing gradually. At the same time the loss curve value is decreasing gradually for training and testing dataset at 2.0 epoch.

**Figure 4:**
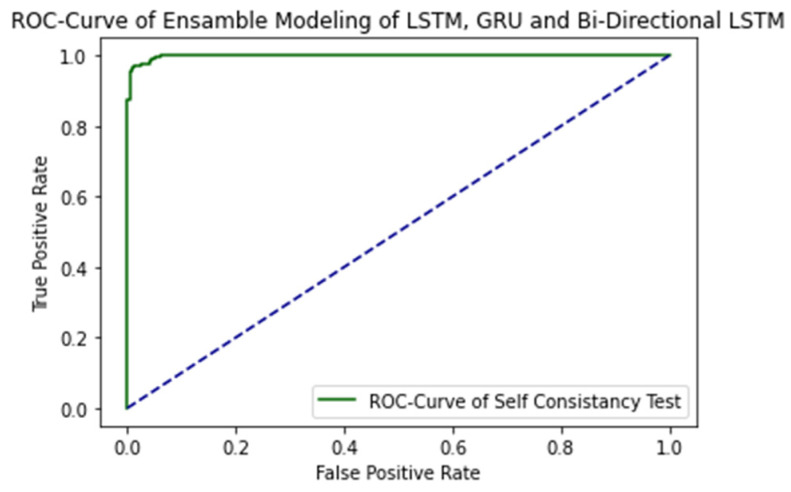
ROC Curve of proposed ensemble learning model with Self Consistency test.

The ROC curve of proposed ensemble learning approach is illustrated in the Figure 4.

The AUC Value is 1.0, which is considered as excellent results according to AUC accuracy classification.

### 2.2. Independent Set Testing

The second testing technique used for the proposed ensemble learning approach is independent set testing. The values are extracted from the misperception matrix used for determining the precision of the model. The independent set test of the proposed model is the basic performance measuring method. From the dataset, 80% of the values are used for training the algorithm and 20% values are used for testing purposes. The results of independent set testing after applying deep learning algorithms are discussed in Table 2.

**Figure 5:**
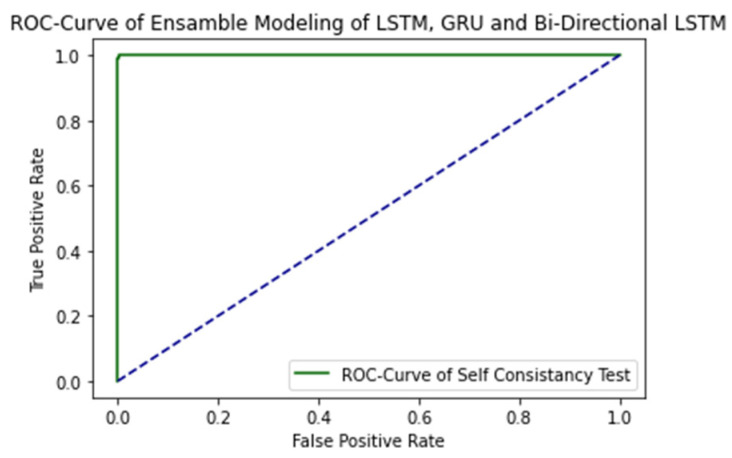
ROC Curve of proposed ensemble learning model with independent set test.

The ROC curve of proposed ensemble learning approach for individual deep learning algorithms is illustrated in the Figure 5.

### 2.3. 10-Fold Cross-Validation Test

In the 10-Fold cross-validation (FCV) technique the data is equally subsamples into 10 groups. Then the training set is divided into 10 partitions and treat each of them in the validation set, training the model and then average generalization performance across the 10-folds to make choices about hyper-parameters and architecture [12].

**Figure 6:**
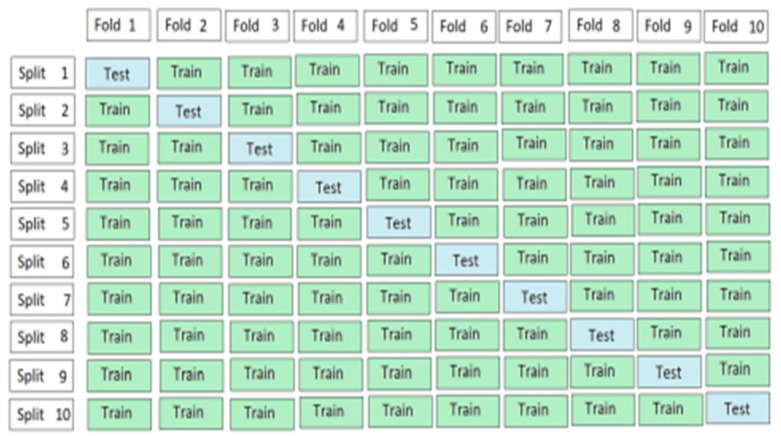
Working process of 10-fold cross-validation.

Figure 6 shows the working process of the 10-fold cross-validation technique.

Table 2 represents the result of proposed ensemble learning approach for individual deep learning algorithms with 10-fold cross-validation technique.

**Figure 7:**
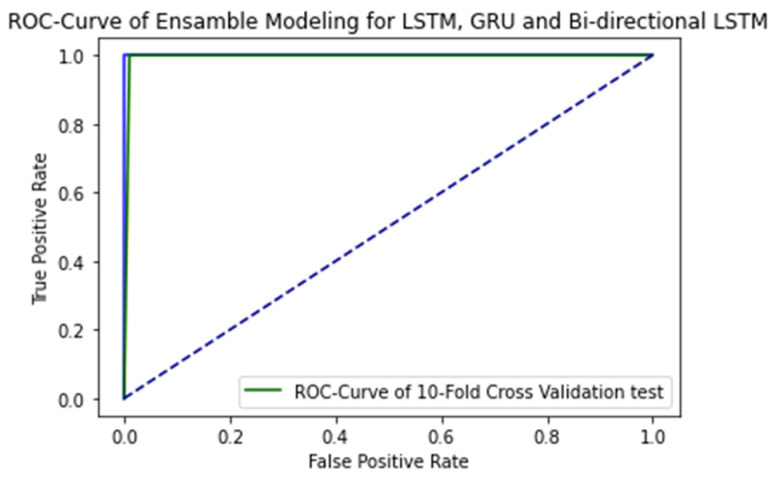
ROC Curve of proposed ensemble learning model with 10-fold Cross validation test.

The ROC curve of proposed ensemble learning approach for individual deep learning algorithm such as LSTM, GRU, and bi-directional LSTM when independent set testing is applied on them is illustrated in the Figure 7.

### 2.4. Results Comparison

The results of ensemble learning approach are compared with its own individual algorithms such as LSTM, GRU, and Bi-directional LSTM in Table 4. Multiple metrics are used for comparison. The independent set test is used for comparison. It is clear from Table 4 that the proposed ensemble learning model improves identification accuracy of the individual deep learning techniques such as LSTM, GRU, and bi-directional LSTM.

Ensemble learning produce better results as compared to simple deep learning algorithms in Table 4. The obtained accuracy through the accuracy is 99.57.

## 3. Analysis and Discussion

Breast adenocarcinoma is the second main cause of death in women worldwide. There are several biological and computational research for the identification and detection of breast adenocarcinoma. In the past studies most of the researchers used some small datasets taken from a small number of hospitals or organizations and applied biological or machine learning algorithms on them for detection with less accuracy and few evaluation matrices.

The proposed ensemble learning approach for individual deep learning including LSTM, GRU, and bi-directional LSTM used the latest generalized big dataset taken from 12 different cohorts for the identification of breast adenocarcinoma. The dataset contains 99 driver genes that cause bread adenocarcinoma where 4127 samples consist of 6170 mutations. In this research the latest dataset for normal and mutated genes sequence of breast adenocarcinoma is used. A similar study is also presented for other types of mutations [17,18] and some testing techniques are also presented in [19,20].

Three different testing techniques including self-consistency test, independent set test, and 10-fold cross validation test are applied on the dataset and the results obtained are 97.6, 99.5 and 98.2 respectively. Therefore, it can be concluded from the results obtained using above mentioned testing techniques that the proposed models are most suitable to achieve high accuracy for cancer prediction. The self-consistency test used the complete dataset for both training and testing phases. Table 1 shows the results obtained using ensemble learning approach with the self-consistency test. The independent set test used 80% of the dataset for training and the remaining 20% for testing. Table 2 shows the results of ensemble learning using independent set test. In the 10-fold cross validation test, 10 equal folds from the whole dataset were created. The proposed ensemble learning model was trained on 9 folds and tested on one-fold, and the same process was repeated. The whole data are used for testing and training. However, shuffled data are provided each time for better learning and, lastly, the average is calculated.

## 4. Materials and Methods

This study proposed a novel ensemble learning approach for deep learning techniques such as LSTM, GRU, and bi-directional LSTM for the detection of breast adenocarcinoma.

### 4.1. Data Acquisition Framework

The dataset is the most crucial part of this research. This dataset is used for training the models, testing the outcome, and validating the results. Data acquisition is the process of collecting reliable and accurate data for research. Data acquisition includes the process of data collection to conduct the research and defining how the data are collected from a valid source [21].

For the proposed study normal gene sequences are extracted from asia.ensambl.org [22] and mutation information of each gene related to breast cancer is extracted from intogen.org (accessed on 18 August 2022) [23]. These normal gene sequences and mutated information are extracted through web scraping code. Web scraping is the process of extracting data from different websites available on the World Wide Web [24]. There is more than 2500 type of cancer genomes involved in mutation [25]. Three types of mutations occur in human genes, namely driver mutation, passenger mutation, and not assist. Driver mutation is the type of mutation in cells that cause cancer. Driver mutation causes abnormal growth of the cells [26]. A mutation that alters the gene sequences but does not cause cancer is known as passenger mutation [27] whereas, not assist gene mutation does not contain any information about the mutation therefore it is not added to this study. The data collection is explained step by step in Appendix A.

**Figure 8:**
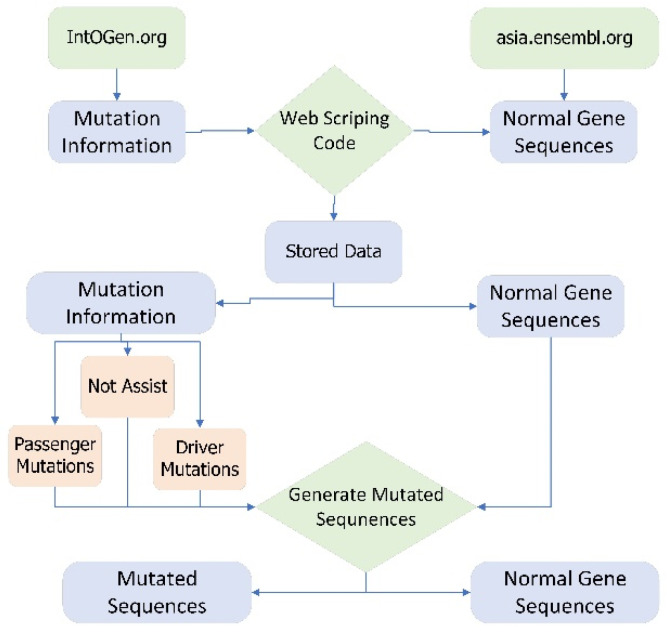
Data acquisition framework of Breast Adenocarcinoma.

A tool named Generate Mutated Sequences (GMS) is created in python that is used to incorporate the mutation information in normal gene sequences and create mutated sequences. Figure 8 shows the data acquisition framework in detail.

From the gene information, mutated gene sequences and normal gene sequences are categorized. For the proposed study 4127 samples are extracted from 99 types of driver genes. The sample dataset is the combination of 12 cohorts of different cancer detection websites which are then combined for this study [23].

The data sample was extracted from every possible combination of age, gender, cancer detection, treatment, and normal person. A total of 6170 mutations is used for training the models, testing the outcome, and validating the results. Driver genes involved in breast adenocarcinoma that cause cancer are shown in Table 5.

**Figure 9:**
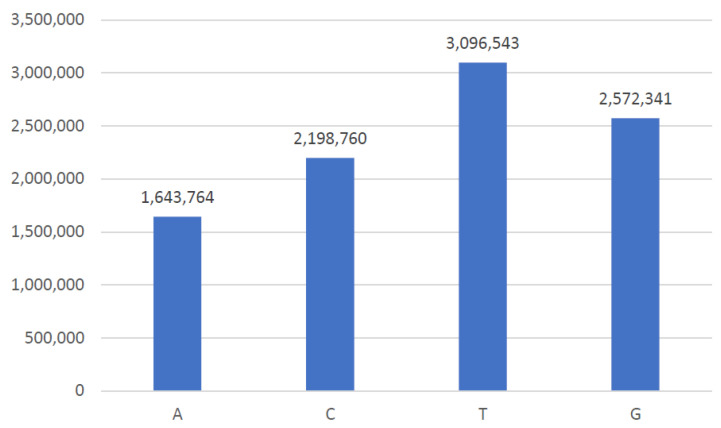
Frequency histogram of bases in Breast Adenocarcinoma gene sequences.

The existence of bases in gene sequences related to breast cancer is explained with the help of the frequency histogram in Figure 9. A total of 99 genes are involved in the progression of breast cancer. Each gene is expressed in a series of bases consisting of nucleotides. Therefore, the dataset contains many nucleotides as expressed with the help of a technique present in Natural Language Processing (NLP) known as a word cloud.

The benchmark dataset for the proposed study is denoted by B, which is defined as

(1) $$B=B^{+}U\;B^{-}\;$$

Here $\;B^{+}$ considered as normal gene sequences while $B^{-}$ is considered as mutated gene sequences that cause cancer and U is the union for both sequences. A balanced dataset is used to provide accurate results [28,29].

### 4.2. Feature Extraction

Feature extraction is used to reduce redundant data. It gives useful features from the available data. Redundancy and irrelevancy are removed after feature extraction. It improves the accuracy and increases the performance of the learning model [30,31,32,33,34,35,36,37,38,39,40,41].

**Figure 10:**
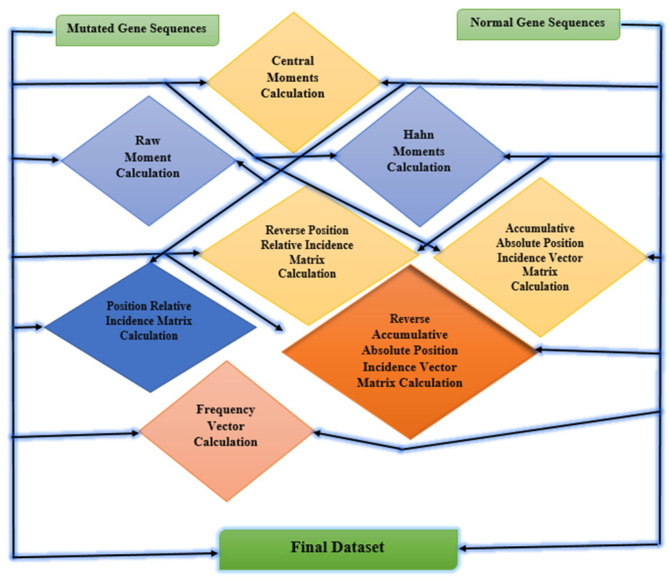
Feature extraction for the breast adenocarcinoma dataset.

The main features of the raw dataset are extracted by feature extraction techniques. Feature extraction is the process of passing data through multiple steps to extract the main features used for model training. It is the most important step in training machine learning algorithms. In feature extraction, the patterns of data are recognized that are further used in the training and testing process performed on data [30,31]. For the proposed study statistical moments are calculated such as Hahn moments, raw moments, and central moments. Other feature extraction techniques are also used including PRIM, RPRIM, AAPIV, and RAAPIV. These feature extraction techniques are applied to mutated gene sequences and normal gene sequences for extracting the main features of data [32]. Figure 10 explains the feature extraction techniques used for the breast cancer dataset.

#### 4.2.1. Hahn Moment Calculation

Hahn moment is used to calculate statistical parameters [42]. Hahn moment is the most important concept in pattern recognition. It calculates the mean and variance in the dataset.

Hahn moments require two-dimensional data [43,44]. Therefore, the genomic sequences are converted into a two-dimensional matrix $G^{\prime }$ of size N×N as in Equation (2).

(2) $$G^{\prime }=\left[\begin{array}{ccc} G_{11} & G_{12\cdots } & G_{1n}\\ G_{21} & G_{22\cdots } & G_{2n}\\ \begin{array}{c} \vdots \\ G_{n1} \end{array} & \begin{array}{c} \vdots \\ G_{n2\cdots } \end{array} & \begin{array}{c} \vdots \\ G_{nn} \end{array} \end{array}\right]$$

Here $G^{\prime }$ defines the gene sequence. The Hahn moments are computed using the value of $G^{\prime }$.

Here each element in is $G^{\prime }$ are the residue of genomic sequences. Statistical moments are calculated in third order. Hahn moments are orthogonal because it takes a square matrix as an input. The Hahn polynomial for the proposed study dataset is calculated by the following Equations (3) and (4).

(3) $$h_{n}^{r,s}\left(A,\;B\right)={\left(B+V-1\right)}_{n}{\left(B-1\right)}_{n}\times \sum_{z=0}^{n}{\left(-1\right)}^{z}\frac{{\left(-n\right)}_{z}{\left(-A\right)}_{z}{\left(2B+r+s-n-1\right)}_{z}}{{\left(B+s-1\right)}_{z}{\left(B-1\right)}_{z}}\;\frac{1}{z!}$$

where r and s are all positive integers. r and s are the predefined constants. n is the order of the moment, and *B* is the size of the data array.

(4) $$C_{xy}=\sum_{j=0}^{G-1}\sum_{i=0}^{G-1}\delta_{xy}h_{x}^{a,b}\left(j,\;B\right)h_{y}^{a,b}\left(j,\;B\right),\;m,\;n=0,\;1,\;2,\ldots ,B-1\;$$

where x+y is the order of the moment, a, b are predefined constants, and $\delta_{xy}$ is an arbitrary element of the square matrix $G^{\prime }$.

For any integer A ∈ [0, B−1] (B is the provided positive integer). These are the adjustable parameters and use to control the shape of polynomials. The Pochhammer symbol is $\left(a\right)k=a\;\cdot \;\left(a+1\right)\cdots \left(a+k-1\right)=\frac{r\left(a+k\right)}{r\left(a\right)}$. Equations (3) and (4) is used to efficiently calculate the normalized Hahn moment of any order. The Hahn moments based unique features are presented by $H_{00},\;H_{01},\;H_{10},\;H_{11},\;H_{02},\;H_{20},\;H_{12},\;H_{21},$ $H_{03}\;\mathrm{and}\;H_{30}$.

#### 4.2.2. Raw Moment Calculation

Raw moment is used for statistics imputation. Imputation is the procedure of replacing the missing data values in a dataset with the most suitable substitute values to keep the facts [45]. The raw moment for the of 2D data with order a+b is expressed by Equation (5) [46].

(5) $$R_{ab}=\sum_{e=1}^{n}\sum_{f=1}^{n}e^{a}f^{b}\delta_{ef}$$

Raw moments are calculated up to order 3. It describes significant information within the sequence such as $R_{00},\;R_{01},\;R_{10},\;R_{02},\;R_{20},\;R_{03,\;}\;\mathrm{and}\;R_{30}$.

#### 4.2.3. Central Moment Calculation

Central moment of feature extraction is used to extract useful features using mean and variance. It is the moment in probability distribution about a randomly selected variable with respect to its random variable mean [42]. The general formula for the central moment calculation for the breast adenocarcinoma dataset is represented by Equation (6).

(6) $$V_{rs}=\sum_{e=1}^{n}\;\sum_{f=1}^{n}{\left(e-\bar{x}\right)}^{r}\;{\left(f-\bar{y}\right)}^{s}\;\delta ef$$

Centroids (r, s) are required to compute the central moments that are visualized as center of data. The unique features from central moments, up to 3rd order, are labeled as $V_{00},\;V_{01},\;V_{10},\;V_{11},\;V_{02},\;V_{20},\;V_{12},\;V_{21},\;V_{03}\;\mathrm{and}\;V_{30}$.

#### 4.2.4. Position Relative Incidence Matrix (PRIM)

PRIM is used for determining the positioning of each gene in the gene sequence of Breast cancer. PRIM formed matrix with the dimension of 30∗30 is shown in Equation (7) [47].

(7) $$R_{PRIM}=\left[\begin{array}{ccc} \begin{array}{cc} R_{1\to 1} & R_{1\to 2\cdots }\\ R_{2\to 1} & R_{2\to 2\cdots } \end{array} & \begin{array}{c} R_{1\to q\cdots }\\ R_{2\to q\cdots } \end{array} & \begin{array}{c} R_{1\to M}\\ R_{2\to M} \end{array}\\ \begin{array}{cc} \vdots & \vdots \\ R_{p\to 1} & R_{p\to 2\cdots } \end{array} & \begin{array}{c} \vdots \\ R_{p\to q\cdots } \end{array} & \begin{array}{c} \vdots \\ R_{p\to M} \end{array}\\ \begin{array}{cc} \vdots & \vdots \\ R_{M\to 1} & R_{M\to 2\cdots } \end{array} & \begin{array}{c} \vdots \\ R_{M\to q\cdots } \end{array} & \begin{array}{c} \vdots \\ R_{M\to M} \end{array} \end{array}\right]$$

Feature scaling lets in every data pattern to participate in detection of ovarian cancer [30]. The indication score of *q*th position nucleotide is determined by the $R_{p\to q}$ with respect to the occurrence of the *p*th nucleotide.

#### 4.2.5. Reverse Position Relative Incidence Matrix (RPRIM)

Reverse Position Relative incidence matrix (RPRIM) also work same as PRIM does but in the reverse sequence. Equation (8) elaborate the calculation of RPRIM for breast cancer dataset.

(8) $$R_{RPRIM}=\left[\begin{array}{ccc} \begin{array}{cc} R_{1\to 1} & R_{1\to 2\cdots }\\ R_{2\to 1} & R_{2\to 2\cdots } \end{array} & \begin{array}{c} R_{1\to q\cdots }\\ R_{2\to q\cdots } \end{array} & \begin{array}{c} R_{1\to M}\\ R_{2\to M} \end{array}\\ \begin{array}{cc} \vdots & \vdots \\ R_{p\to 1} & R_{p\to 2\cdots } \end{array} & \begin{array}{c} \vdots \\ R_{p\to q\cdots } \end{array} & \begin{array}{c} \vdots \\ R_{p\to M} \end{array}\\ \begin{array}{cc} \vdots & \vdots \\ R_{M\to 1} & R_{M\to 2\cdots } \end{array} & \begin{array}{c} \vdots \\ R_{M\to q\cdots } \end{array} & \begin{array}{c} \vdots \\ R_{M\to M} \end{array} \end{array}\right]$$

#### 4.2.6. Accumulative Absolute Position Incidence Vector (AAPIV)

The frequency matrix gives the information about the incidence of genes in the gene sequence. AAPIV gives the information related to the different compositions of nucleotides in the gene sequences. The relative positioning of the nucleotides in cancerous gene sequences is observed out by using AAPIV [46]. The relative gene sequences of breast adenocarcinoma are illustrated with the help of Equation (9).

(9) $$K=\left\{\lambda_{1},\lambda_{2},\ldots \lambda_{n}\right\}$$

where $\lambda_{n}$ is from gene sequence having ‘n’ total nucleotides, which can be calculated using Equation (10).

For any *i*th component,

(10) $$\lambda_{i}=\sum_{k=1}^{n}\beta_{k}$$

where $\beta_{k}$ is the position of the $i^{th}$ nucleotides.

#### 4.2.7. Reverse Accumulative Absolute Position Incidence Vector (RAAPIV)

RAAPIV work the same as AAPIV works but in the reverse order. The equation for RAAPIV is as follows.

(11) $$\lambda =\left\{n_{1},n_{2},\ldots n_{m}\right\}$$

#### 4.2.8. Frequency Vector Calculation

A dataset contains thousands of data records with different attributes for each record. A frequency matrix is used to represent the sequence of genes that combine to form a gene sequence. The distribution of each gene in the gene sequence of breast adenocarcinoma is utilized to form a frequency distribution vector. It is represented by Equation (12).

(12) $$\alpha =\left\{\varepsilon_{1},\varepsilon_{2},\ldots \varepsilon_{n}\right\}$$

Here is the frequency of the genes in the breast adenocarcinoma gene sequence. The frequency vector is calculated by the Equation (13).

(13) $$FV=\left\{{\;f}_{1\;},{\;f}_{2},{\;f}_{3\;}\ldots {\;f}_{N\;}\right\}$$

Here ${\;f}_{1\;}$ to ${\;f}_{N\;}$ indicates the frequency of each gene in the gene sequence.

### 4.3. Algorithm for Predictive Modeling

For the proposed study, a deep neural network with multiple layers is used for the detection of breast adenocarcinoma. Deep learning has a huge impact on the recognition, detection, prediction, and diagnosis of different types of cancer, forecasting, detection systems, and many other complex problems. A deep neural network model consists of multiple layers including an input layer, an output layer, pooling layer, dense layer, and dropout layer with fully connected layers at the top. Each of the layers takes the input from the previous layer and processes those input features. The learning features inside these layers are the algorithms that learn from the layers and train themselves using different learning procedures [48].

This study uses three different types of deep learning RNN algorithms including Long short-term memory (LSTM), Gated recurrent units (GRU), and bi-directional LSTM. These algorithms use three evaluation methods that are a self-consistency test, independent set test, and a 10-fold cross validation test for the identification of breast adenocarcinoma.

#### 4.3.1. Long Short-Term Memory Network (LSTM)

**Figure 11:**
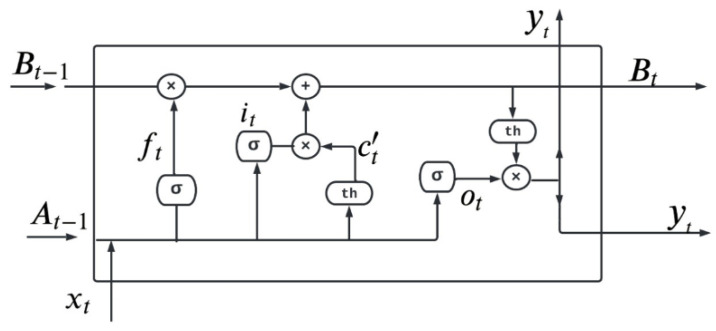
LSTM cell structure for breast adenocarcinoma.

LSTM is the first deep learning algorithm used in this process. LSTM is used to resolve the vanishing gradient problem in the neural network. Vanishing gradient is a problem in which the lost function approximately approaches zero and makes the neural network hard for training [49,50]. LSTM is used to address short-term and vanishing gradient problems in RNN [51]. It increases the memory of the RNN model. LSTM is a gated process all the information in LSTM is read, stored, and written with the help of these gates. These gates are of three types known as input gate, forget gate, and output gate [52]. The gate in LSTM is responsible for learning the regulation of some information from one gate to another gate. Therefore, different activation functions are utilized in every gate [53,54]. Figure 11 explains the structure of a simple gated cell used in the LSTM technique for the detection of breast cancer.

In Figure 11 $x_{t}\;$ is the input at specific time and $y_{t}$ is the output at specific time t. $f_{t\;}$ represents forget gate, $O_{t}\;$ and $i_{t}\;$ represent output gate and input gate, respectively. Every cell of LSTM has three inputs $x_{t},\;A_{t-1},\;B_{t-1}\;$and has two outputs as $b_{t}\;$and $h_{t}$. Equations (14)–(19) explain LSTM.

(14) $$i_{t}=\sigma \;\left(y_{t}U^{i}+A_{t-1}\;W^{i}\right)$$

(15) $$f_{t}=\sigma \;\left(y_{t}U^{f}+A_{t-1}\;W^{f}\right)$$

(16) $$o_{t}=\sigma \;\left(x_{t}U^{o}+A_{t}\;W^{o}\right)\;$$

(17) $${B_{t}}^{\prime }=tanh\;\left(x_{t}U^{c}+A_{t-1}\;W^{c}\right)$$

(18) $$B_{t}=\sigma \;\left(f_{t}*\;B_{t-1}+i_{t}*{B_{t}}^{\prime }\;\right)$$

(19) $$y_{t}=tanh\;\left(B_{t}\right)\;*\;O_{t}\;$$

In the equations $x_{t}$ is the input, $A_{t-1}$ is the previous data cell output, $B_{t-1}$ is the previous cell memory, $B_{t}$ is the current cell memory. Here W and U are the weights for the forget, input, and output gate, respectively.

The data enter directly into the embedding layer form the input layer. The embedding layer is the first hidden layer of LSTM. It consists of input dimension, output dimension, and input length. The output of the embedding layer is denoted by the Equation (20) [55].

(20) $$E_{out}=V_{i}*\;X_{i}$$

In the equation $E_{out}\;$is the output of the embedding layer, $V_{i}$ is the parameter between the input layer and embedding layer while $X_{i}$ is the one hot vector if it is filed. One hot vector is used to differentiate data from each other. Data from the embedding layer are entered into the LSTM layer where they pass from the LSTM gates. The embedding is used to convert the input into a fixed length. The input length is converted to 64. An LSTM layer of 128 neurons is added. The dropout layer prevents the model from overfitting and the dense layer connects all the input from the layer and passes to the output layer. Two dropout layers are used in this model to overcome model overfitting. One dense layer is used as a hidden layer with 10 neurons. Stochastic Gradient Descent (SGD) is used as an optimizer in LSTM layer. Sigmoid is used as an activation function. Sparse Categorical Cross Entropy (SCCE) is used to minimize the loss in training the proposed model.

#### 4.3.2. Gated Recurrent Unit (GRU)

**Figure 12:**
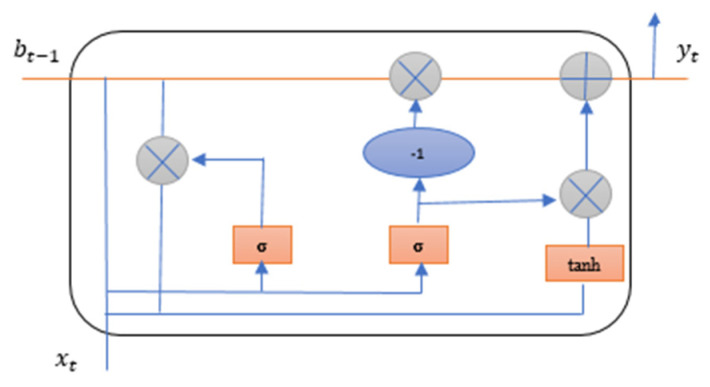
GRU cell structure for breast adenocarcinoma.

The second deep learning method used for the proposed study is Gated Recurrent Unit (GRU) method. GRU uses fewer gates than LSTM and works in the same way. The results obtained from GRU are better than LSTM due to the smaller number of gates and parameters. GRU use only two gates reset gate and the update gate in the cell [52]. The reset gate of GRU decides how much past information is neglected and update gate decides how much past information is to be used. GRU takes less computational time than LSTM [56]. Figure 12 explains the GRU cell structure used in the identification of breast adenocarcinoma.

The following Equations (21)–(24) explains GRU.

(21) $$r_{t}=\sigma \;\left(x_{t}U^{r}+B_{t-1}\;W^{r}\right)$$

(22) $$z_{t}=\sigma \;\left(x_{t}U^{z}+B_{t-1}\;W^{z}\right)$$

(23) $${h_{t}}^{\prime }=tanh\;\left(r_{t}*B_{t-1}\;U+x_{t}W\right)$$

(24) $$y_{t}=\left(\;1-z_{t}\right)*{B_{t}}^{\prime }+z_{t}*\;B_{t-1}\;)\;$$

In the Equations (21) and (22) $r_{t}\;$represents reset gate and $z_{t}$ is the update gate.

The proposed model has one embedding layer to convert the input into a vector of fixed word length of 64. The second layer is GRU with 256 neurons and a simple RNN Layer with 128 neurons. Two dropout layers are added with 30 percent to prevent overfitting. One dense layer is added at the end with 10 neurons. Stochastic Gradient Descent (SGD) is used as an optimizer in GRU layer. Sigmoid is used as an activation function. Sparse Categorical Cross Entropy (SCCE) is used to minimize the loss in training the proposed model.

#### 4.3.3. Bi-Directional LSTM

Lastly, the deep learning technique used in the proposed study is bi-directional LSTM [57]. A bi-directional LSTM uses two LSTM cells, one in the forward direction and one in the backward direction, connected with a single output.

The proposed Model has one embedding layer to convert the input into fixed vectors of fixed word length of 64. Two bi-directional layers with 128 and 64 neurons in both directions are added. Three dropout layers are added with 30 percent to prevent from overfitting. One dense layer is used with 64 neuron and one dense layer is added at the end with 10 neurons. Stochastic Gradient Descent (SGD) is used as an optimizer in GRU layer. Sigmoid is used as an activation function. Sparse Categorical Cross Entropy (SCCE) is used to minimize the loss in training the proposed model.

Unlike LSTM and GRU, Bi-directional LSTM did not need any past knowledge for the prediction it learns by itself through moving in forward and backward direction that’s why the result of Bi-directional LSTM is better than LSTM and GRU [58].

#### 4.3.4. Ensemble Learning Models

Ensemble learning model uses a divide-and-conquer approach. It is used to improve the accuracy of an individual base learners and then compile the whole model. Multiple base learners are combined to achieve the best results [59]. Each base learner learns different features from data chunks obtained using bootstrap technique, generates some results, and combines them. Then again, the data chunks feed to the model. In this way the patterns hidden in the datasets are learned by the model. Ensemble learning is an adaptable approach and shows better accuracy as compared to simple machine learning algorithms. This is due to the bootstrap technique, which allows feature replacement and row replacement techniques, and the model learns using all the possible data combinations. This also results in overcoming the overfitting issues. The popular ensemble learning model types are bagging [60], boosting [61] and stacking [62]. The aim of all these models is to obtain good accuracy.

**Figure 13:**
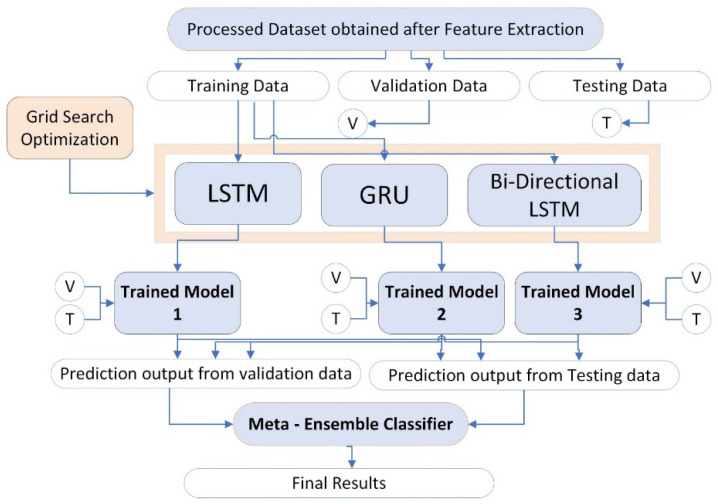
Proposed methodology of Ensemble Learning Model of LSTM, GRU, and bi-directional LSTM.

This study improves the performance of individual deep learning models such as LSTM, GRU, and Bi-directional LSTM with the help of an ensemble learning approach. The processed dataset is divided into three groups such as training set, validation set, and test set. The validation set is denoted by V whereas, the test set is denoted by T. The training set is given as input to each individual deep learning model which are LSTM, GRU, and bi-directional LSTM. The grid search optimization technique is also applied to get search ranges and the optimum values for proposed ensemble learning model parameters. Trained learning models are generated for each individual deep learning model by the name trained model1, trained model2, and trained model3 for LSTM, GRU, and Bi-directional LSTM respectively as shown in Figure 13.

All the trained models are tested on both validation and testing sets. Lastly, final improved results are obtained by an ensemble learning model as shown in results section.

(25) $$gp,i=\sum_{n=1}^{N}\omega_{n}f_{n,i}$$

Weights are assigned to the individual deep learning model to construct the ensemble learning prediction in the equation. Here $\omega_{n}\;\left(n=1,\;2,\;\ldots ,\;N\right)$ is the weight assigned to each individual deep learning model, $f_{n,\;i}$ represents the prediction of each individual deep learning model whereas, n is for the ith observation.

For each deep learning technique these testing techniques are applied in 10 epochs (10 feed-forward and feed backward paths). In each iteration for testing the model calculates its AUC, precision, F1 score, recall, Cohen’s kappa, specificity, sensitivity, Mathew’s correlation coefficient, loss, and accuracy. The following are the mathematical equations used to calculate the algorithms’ results [63,64,65,66]. The Equations (26)–(29) explain the formulae to calculate sensitivity, specificity, accuracy, and Mathews Correlation Coefficient (MCC) respectively.

(26) Sensitivity=TP/(TP+FN)

(27) Specificity=TN/(TN+FP)

(28) Accuracy=(TP+TN)/(TP+FP+FN+TN)

(29) $$\mathrm{MCC}=\frac{\left(TP\;X\;TN\right)-\left(FP\;X\;FN\right)}{\sqrt{\left(TP+FP\right)\left(TP+FN\right)\left(TN+FP\right)\left(TN+FN\right)}}$$

In these equations:

TN = All the true negative values

TP = All the true positive values from the dataset

FN = False negative values

FP = False positive values

In the above equations, sensitivity refers to the ability to predict the count that truly identify the breast adenocarcinoma. Specificity refers to the ability to predict the count that truly identify the absence of breast cancer. TP+FN are all subjects with given condition. While TN+FP are the subjects without the given conditions. TP+FP is the total number of subjects with positive results and TN+FN is the subjects with the negative results.

## 5. Conclusions and Future Work

Breast adenocarcinoma is the most common cancer in women. Therefore, a proposed ensemble learning approach with individual deep learning techniques that includes LSTM, GRU, and bi-directional LSTM is developed for the early detection of breast adenocarcinoma. Normal gene sequences are obtained from asia.ensembl.org and mutation information is obtained from IntOgen.org. Mutated sequences are generated by incorporating mutation information into normal gene sequences as depicted in Figure 8. A feature extraction technique is used to obtain useful features from the normal and mutated gene sequences. The feature extraction also converts the data to numeric format which is ready for training and testing. Multiple feature extraction techniques used in this study can be seen in Figure 10. The proposed ensemble learning approach obtained 99.57% accuracy. Multiple testing techniques such as self-consistency test, independent set test, and 10-fold cross validation tests are applied to check the performance of the model. The ensemble learning approach has good performance in an independent set test as shown in Table 2. All the results are compared in Table 4. Therefore, it can be concluded from the results that the proposed ensemble learning approach performs with high accuracy for the identification of breast adenocarcinoma. The results of Accuracy, AUC, Loss, Sensitivity, Specificity, and Mathew’s correlation coefficient of the independent test, self-consistency test and 10-fold cross-validation test are shown in Table 1, Table 2 and Table 3. Lastly, the results authenticate that the proposed ensemble learning model using deep learning classifiers can be utilized efficiently for any cancer detection.

In future this technique can be used further for the detection of other types of cancer as well. Furthermore, this technique can be beneficial to detect the incidence of other life-threatening diseases using genes.

## Figures and Tables

**Table 1:** Results of Proposed Ensemble Learning Model with Self Consistency Test.

Evaluation Matrices	Values	Evaluation Matrices	Values
Accuracy (%)	97.65	Precision (%)	97.65
Sensitivity (%)	97.81	Recall (%)	97.65
Specificity (%)	97.50	F1 Score (%)	97.65
MCC	0.95	Cohens Kappa (%)	95.31
AUC	1.00	Training Accuracy (%)	78.29
Training Loss	0.3649	Testing Accuracy (%)	78.51

**Table 2:** Results of Proposed Ensemble Learning Model with Independent set Test.

Evaluation Matrices	Values	Evaluation Matrices	Values
Accuracy (%)	99.57	Precision (%)	99.57
Sensitivity (%)	99.50	Recall (%)	99.57
Specificity (%)	99.63	F1 Score (%)	99.57
MCC	0.99	Cohens Kappa (%)	99.14
AUC	1.00	Training Accuracy (%)	99.79
Training Loss	0.2027	Testing Accuracy (%)	99.82

**Table 3:** Results of proposed ensemble learning model with 10-fold cross validation test.

Evaluation Matrices	Values	Evaluation Matrices	Values
Accuracy (%)	98.26	MCC	0.9852
Sensitivity (%)	98.02	AUC	0.99
Specificity (%)	98.50		

**Table 4:** Comparison of ensemble learning with individual deep learning techniques.

Evaluation Matrices	Ensemble Learning Approach	LSTM	GRU	Bi-Directional LSTM
Accuracy (%)	99.57	99.02	97.12	96.51
Sensitivity (%)	99.50	98.89	96.94	96.32
Specificity (%)	99.63	99.14	97.31	96.69
MCC	0.99	0.98	0.94	93.02
AUC	1.00	1.00	1.00	1.00
Training Loss	0.2027	0.0235	0.1199	0.2122
Precision (%)	99.57	99.02	97.12	96.51
Recall (%)	99.57	99.02	97.12	96.51
F1 Score (%)	99.57	99.02	97.12	96.51
Cohens Kappa (%)	99.14	98.04	94.25	93.02
Training Accuracy (%)	99.79	99.30	95.60	97.70
Testing Accuracy (%)	99.82	99.44	97.43	96.94

**Table 5:** Symbols of genes involved in breast adenocarcinoma.

Gene	Mutation	Gene	Mutation	Gene	Mutation
TP53	846	KMT2C	205	ERBB4	43
GATA3	63	CDI	176	MDM4	14
ESR1	129	PTEN	105	GATA1	15
AKT1	88	NCOR1	89	USP6	19
FOXA1	72	TBX3	54	EGFR	45
NF1	85	ERBB2	83	MEN1	28
RB1	60	CFFB	64	GNAS	29
SF3B1	56	KMT2D	99	KDM6A	30
FAT3	112	ERBB3	55	FAT4	78
PREX2	73	CTFC	47	KAT6B	37
LRP1B	114	RUNX1	37	JAK2	19
ATM	64	SPEN	74	ALK	33
FGFR2	37	BRCA1	49	BAP1	25
CASP8	28	FBXW7	29	CUX1	29
BRCA2	52	PTPRD	64	KLF4	8
MYH11	59	RGS7	32	FAT1	61
KRAS	15	NCOA1	21	DDX3X	23
MYH9	59	ABL2	31	NONO	9
EPHA3	31	NCOR2	44	MTOR	57
AFF3	37	ETV5	16	ASXL1	36
BRAF	22	ELN	26	MYOSA	19
ZXBD	18	NTRK1	26	POLD1	18
SALL4	17	SMAD2	17	PLAG1	15
EPAS1	25	RHPN2	18	NIN	44
SMAD4	17	MAX	9	NUMA1	33
HAS	10	ZFHX3	72	CLTC	31

## Data Availability

Data can be received from author Asghar Ali Shah via alishahsadiq@gmail.com.

## Appendix A

Databases used in this study

This Appendix A is used to discuss and explain the databases used in this study. Normal gene sequences are extracted from asia.ensambl.org [16] and mutation information of each gene related to breast adenocarcinoma is extracted from intogen.org [17]. These normal gene sequences and mutation information are extracted through web scraping code. Web scraping is the process of extracting data from different websites available on the World Wide Web [18].

Step by step process of extracting dataset

Go to “https://intogen.org/search” (accessed on 10 September 2022). Click on breast adenocarcinoma in the circle under “IntOGen Samples”. Click on the table tab under “Mutational cancer driver genes”. All 99 genes are listed here. Click on first gene named “TP53”. Click on table tab under “Observed mutations in tumors”. All mutation information is present here for this single gene. You can see mutation information related to other genes by clicking each gene. At the write top of this page is written “Gene details”. Under “Gene details” there is written “Ensembl id”. This is the id of normal gene sequences related to the selected gene. If you click on this link, you will go to http://asia.ensembl.org/ (accessed on 10 September 2022) where you will find normal gene sequences. Click on this link and then click on the sequence on the left panel. Normal sequences will be shown in the main page and a download link is also available to download this normal gene sequence. repeat this process 99 times for each gene to download the whole dataset of Breast adenocarcinoma.
